# Supplementary material for: An Artificial Intelligence Approach to Bloodstream Infections Prediction
Source: J Clin Med. 2021 Jun 29;10(13):2901. doi: 10.3390/jcm10132901 (PMC8268222; doi:10.3390/jcm10132901)
Supplement: Supplementary file 1 [file jcm-10-02901-s001.zip › jcm-1247778-SI.pdf]

Supplementary Materials

Table S1. The relevant studies in predicting BSIs.

References	Study Aims	Study Population/ Dataset	Preprocessing/ Statistical Analysis	Machine Learning / Deep Learning Algorithms	Evaluation
Lee et al. 2019 [1]	Early Detection of Bacteraemia with an Artificial Neural Network Approach	general wards, Adults (age > 18 years)/ Gangnam Severance Hospital, South Korea	Student's t-test, chi-squared test, and internal cross-validation	Artificial neural network, multi-layer perceptron, random forest, and support vector machine	AUROC, Sensitivity, Specificity
Roimi et al. 2020 [2]	A machine-learning (ML) algorithm for bloodstream infection in ICU	ICU, Adults (age > 18 years)/ Beth Israel Deaconess Medical Center, USA; Rambam Healthcare Campus, Israel	Student's t-test, chi-squared test, and ten-fold cross-validation	Random forest, Extreme gradient boosting and logistic regression	AUROC Sensitivity, Specificity, positive predicted value, negative predicted value
Park et al. 2020 [3]	Detection of Bacteremia using Recurrent Neural Network	General surgeries, Asan Medical Center, South Korea	Regular Expression for ICD-9-CM, Embedding method	Recurrent Neural Network	AUROC, AUPRC, Sensitivity, Specificity
Mahmoud et al. 2021 [4]	Developing Machine-Learning Prediction Algorithm for Bacteremia	Adults (age > 14 years)/ King Abdulaziz Medical City, Saudi Arabia	Univariate and multivariate binary logistic regression	Random Forest, Logistic Regression, Decision Trees, Neural Networks, and support vector machine	Accuracy, Precision, Specificity, Sensitivity, AUROC
Tsai et al. 2021 [5]	Using machine learning to build a model that could predict bacteremia in febrile children	Febrile children at the pediatric emergency department/ Kaohsiung Chang Gung Memorial Hospital, Taiwan	Student t tests, Fisher's exact test, chi-squared test, univariate and multivariate binary logistic regression.	logistic regression and support vector machines	AUROC, Sensitivity, Specificity

Table S2. The distribution of pathogens among the BSIs.

Pathogens	Numbers of Patients
Acinetobacter baumannii	159
acinetobacter spp.	33
Bacteroides spp.	16
Candida	121
Chryseobacterium spp.	38
Enterobacter spp.	92
Enterococcus	106
E. Coli	70
Klebsiella pneumoniae	283
Proteus spp.	14
Pseudomonas aeruginosa	121
Serrratia marcescens	53
Staphylococcus aureus	127
Staphylococcus spp.	133
Others	112
**SUM**	**1478**

Table S3. Patient demographics of study population in each dataset.

	**Training dataset** n **= 3018**		**Validation dataset** n **= 1006**		**Testing dataset** n **= 1006**		***p*-Value**	
	BSI $n = 884$	Non-BSI $n = 2134$	BSI $n = 308$	Non-BSI $n = 698$	BSI $n = 286$	Non-BSI $n = 720$	BSI	Non-BSI
**Basic characteristics**								
Age, yesrs	67.40 ± 14.49	65.72 ± 16.29	66.61 ± 15.16	65.56 ± 16.41	68.79 ± 15.00	66.69 ± 15.88	0.186	0.319
Sex (male)	616 (69.68%)	1412 (66.17%)	220 (71.43%)	451 (64.61%)	204 (71.33%)	451 (62.64%)	0.782	0.217
Charlson comorbidity index	2.35 ± 1.41	2.19 ± 1.42	2.38 ± 1.44	2.21 ± 1.45	2.32 ± 1.39	2.16 ± 1.46	0.880	0.848
APACHE II score	26.52 ± 6.06	25.45 ± 6.17	26.22 ± 6.12	25.39 ± 6.21	26.58 ± 6.25	25.37 ± 5.86	0.719	0.939
**Divisions**							0.983	0.519
MICU	369 (41.74%)	838 (39.27%)	126 (40.91%)	277 (39.68%)	120 (41.96%)	294 (40.83%)		
SICU	296 (33.48%)	483 (22.63%)	98 (31.82%)	152 (21.78%)	95 (33.22%)	168 (23.33%)		
CCU	146 (16.52%)	282 (13.21%)	59 (19.16%)	99 (14.18)	47 (16.43%)	94 (13.06%)		
CV	68 (7.69%)	163 (7.64%)	27 (8.77%)	61 (8.74%)	20 (6.99%)	65 (9.03%)		
CVS	78 (8.82%)	119 (5.58%)	32 (10.39%)	38 (5.44%)	27 (9.44%)	29 (4.03%)		
NICU	73 (8.26%)	531 (24.88%)	25 (8.11%)	170 (24.35%)	24 (8.39%)	164 (22.77%)		
NEURO	13 (1.47%)	77 (3.61%)	5 (1.62%)	32 (4.58%)	7 (2.45%)	32 (4.44%)		
NS	60 (6.79%)	454 (21.27%)	20 (6.49%)	138 (19.77%)	17 (5.94%)	132 (18.33%)		
**The etiology for ICU admission**							0.974	0.805
Scheduled surgery	109 (5.44%)	33 (3.93%)	9 (3.09%)	45 (6.86%)	7 (2.55%)	38 (5.65%)		
Emergent surgery	54 (2.70%)	14 (1.67%)	7 (2.41%)	17 (2.59%)	10 (3.64%)	11 (1.63%)		
NS surgery, scheduled	33 (1.65%)	3 (0.36%)	0 (0.00%)	5 (0.76%)	1 (0.36%)	8 (1.19%)		
NS surgery, emergent	179 (8.94%)	13 (1.55%)	5 (1.72%)	64 (9.76%)	4 (1.45%)	53 (7.88%)		
Acute respiratory failure	470 (23.48%)	196 (23.33%)	66 (22.68%)	162 (24.70%)	63 (22.91%)	173 (25.71%)		
Pneumonia	152 (7.59%)	56 (6.67%)	17 (5.84%)	39 (5.95%)	19 (6.91%)	44 (6.54%)		
Sepsis, non-pneumonia	114 (5.69%)	76 (9.05%)	25 (8.59%)	35 (5.34%)	24 (8.73%)	34 (5.05%)		
Acute cardiac conditions	235 (11.74%)	97 (11.55%)	36 (12.37%)	73 (11.13%)	31 (11.27%)	72 (10.70%)		
Acute neurological conditions	88 (4.40%)	15 (1.79%)	7 (2.41%)	23 (3.51%)	7 (2.55%)	30 (4.46%)		
Pulmonary embolism	0 (0%)	0 (0%)	0 (0%)	0 (0%)	0 (0%)	0 (0%)		
Acute renal conditions	24 (1.20%)	5 (0.60%)	6 (2.06%)	6 (0.91%)	3 (1.09%)	14 (2.08%)		
PostPCI	244 (12.19%)	168 (20.00%)	56 (19.24%)	85 (12.96%)	52 (18.91%)	83 (12.33%)		
OHCA/INCA	10 (0.50%)	4 (0.48%)	1 (0.34%)	3 (0.46%)	1 (0.36%)	3 (0.45%)		
Others	14 (0.70%)	9 (1.07%)	2 (0.69%)	6 (0.91%)	1 (0.36%)	7 (1.04%)		
**Outcomes**	276 (13.79%)	151 (17.98%)	54 (18.56%)	93 (14.18%)	52 (18.91%)	103 (15.30%)		
ICU-stay, days								
Hospital-stay, days	33.43 ± 24.45	22.03 ± 17.12	35.23 ± 26.51	22.46 ± 14.85	34.94 ± 27.01	22.11 ± 13.98	0.463	0.829

Table S4. Logistic regression analysis for crude and adjusted odds ratio.

Variables	Crude odds ratio	95% CI	*p*-value	Adjusted odds ratio	95% CI	*p*-value
**Basic characteristics**						
Gender	0.787	(0.690, 0.898)	<0.001	0.749	(0.649, 0.865)	<0.001
Age	1.007	(1.003, 1.011)	0.001	1.005	(1.000, 1.010)	0.036
APACHE II Score	1.028	(1.018, 1.039)	<0.001	1.004	(0.993, 1.016)	0.445
Comorbidity Score	1.083	(1.038, 1.129)	<0.001	0.980	(0.932, 1.029)	0.415
**Vital Sign**						
Temperature	0.733	(0.645, 0.833)	<0.001	0.918	(0.807, 1.044)	0.194

SBP	0.989	(0.985, 0.993)	<0.001	0.996	(0.991, 1.001)	0.148
DBP	0.988	(0.982, 0.993)	<0.001	1.002	(0.994, 1.010)	0.563
GCS	0.960	(0.943, 0.978)	<0.001	0.989	(0.968, 1.010)	0.287
Heart-rate	1.009	(1.005, 1.013)	<0.001	1.003	(0.999, 1.008)	0.154
Respiratory rate	1.010	(0.993, 1.027)	0.249	0.994	(0.975, 1.013)	0.543
Albumin	0.522	(0.463,0.588)	<0.001	0.715	(0.624, 0.819)	<0.001
Alkaline phosphatase	1.001	(1.000, 1.001)	<0.001	1.000	(1.000, 1.000)	0.834
BUN	1.009	(1.007, 1.011)	<0.001	1.006	(1.004, 1.009)	<0.001
Creatinine	1.079	(1.047, 1.111)	<0.001	0.967	(0.923, 1.012)	0.149
CRP	0.998	(0.991, 1.006)	0.704	0.995	(0.987, 1.004)	0.288
Glucose	1.000	(0.999, 1.001)	0.574	1.000	(0.999, 1.000)	0.319
HCO3-A	0.962	(0.949, 0.976)	<0.001	0.982	(0.966, 0.997)	0.023
Hematocrit	0.937	(0.925, 0.950)	<0.001	0.991	(0.973, 1.011)	0.380
Hemoglobin	0.785	(0.754, 0.818)	<0.001	0.937	(0.883, 0.995)	0.034
Sodium(K)	0.887	(0.809, 0.973)	0.011	0.886	(0.800, 0.982)	0.022
Na	0.990	(0.982, 0.999)	0.025	0.981	(0.972, 0.991)	<0.001
PH	0.895	(0.325, 2.462)	0.830	3.001	(0.931, 9.675)	0.066
Platelet count	0.995	(0.995, 0.996)	<0.001	0.997	(0.996, 0.998)	<0.001
PO2-A	1.000	(0.998, 1.001)	0.414	1.000	(0.999, 1.001)	0.930
Prothrombin time	1.036	(1.022,1.049)	<0.001	1.016	(1.004, 1.029)	0.010
WBC	0.992	(0.983, 1.001)	0.066	0.996	(0.986, 1.005)	0.358
Lactate	1.004	(1.000,1.008)	0.081	1.004	(0.999, 1.008)	0.094
**Clinical Information**						
ICU day to BC, days	1.025	(1.021, 1.030)	<0.001	1.022	(1.016, 1.027)	<0.001
Central venous catheter	1.000	(1.000, 1.000)	<0.001	1.000	(1.000, 1.000)	0.125
ENDO	1.000	(1.000, 1.000)	<0.001	1.000	(1.000, 1.000)	0.891
FOLEY	1.000	(0.999, 1.000)	<0.001	1.000	(1.000, 1.000)	0.920

Table S5. Vital-signs values assumed to be plausible.

Temperature (°C)	25-45
SBP (mmHg)	0~300
DBP (mmHg)	0~150
Heart rate (bpm)	0~200
Respiratory rate (breath/min)	0~50

Table S6. The missing values of features.

Features	Percent of missing values of the features during the seven days prior BC sampling
**Basic characteristics**	
Apache II Score	15.55%
Comorbidity Score	0.00%
**Vital Sign**	
Temperature (° C)	0.00%
SBP (mmHg)	0.00%
DBP (mmHg)	0.00%
GCS	0.00%
Heart-rate (bpm)	0.00%
Respiratory rate (breath/min)	0.00%

**Laboratory**	
Albumin (g/dL)	15.32%
Alkaline phosphatase (U/L)	39.23%
BUN (mg/dL)	4.83%
Creatinine (mg/dL)	2.11%
CRP (mg/dL)	58.65%
Glucose (mg/dL)	58.71%
HCO3-A (mmol/L)	19.20%
Hematocrit (%)	15.59%
Hemoglobin (g/dL)	0.04%
Potassium (K) (mEq/L)	0.06%
Na (mEq/L)	0.06%
pH (blood gas)	19.20%
Platelet count (/UL)	0.15%
PO2-A (mmHg)	19.20%
Prothrombin time (PT-P) (s)	26.45%
WBC (/UL)	0.06%
Lactate (mg/dL)	22.29%
**Clinical Information**	
ICU day to BC, days	0.00%
Central venous catheter	0.00%
ENDO	0.00%
FOLEY	0.00%

SBP, systolic blood pressure; DBP, diastolic blood pressure; GCS, Glasgow Coma Scale; BUN, blood urea nitrogen; CRP, C-reactive protein; WBC, white blood cell.

Table S7. The LR, SVM, and MLP models performances of different cut-off threshold.

Algorithms	Cut-off threshold	Sensitivity	Specificity	Precision	True Positive	True Negative	False Positive	False Negative
LR	0.3	91.6%	22.1%	31.8%	262 (26.0%)	159 (15.8%)	561 (55.8%)	24 (2.4%)
	0.4	78.0%	44.4%	35.8%	223 (22.2%)	320 (31.8%)	400 (39.8%)	63 (6.2%)
	0.41	77.3%	47.5%	36.9%	221 (21.9%)	342 (34.0%)	378 (37.6%)	65 (6.5%)
	0.5	61.5%	64.4%	40.7%	176 (17.5%)	464 (46.1%)	256 (25.5%)	110 (10.9%)
	0.53	56.3%	69.2%	42.0%	161 (16.0%)	498 (49.5%)	222 (22.1%)	125 (12.4%)
	0.6	43.4%	81.1%	47.7%	124 (12.3%)	584 (58.1%)	136 (13.5%)	162 (16.1%)
	0.7	21.3%	93.9%	58.1%	61 (6.0%)	676 (67.2%)	44 (4.4%)	225 (22.4%)
SVM	0.3	85.0%	34.7%	34.1%	243 (24.2%)	250 (24.9%)	470 (46.7%)	43 (4.2%)
	0.4	69.2%	59.6%	40.5%	198 (19.7%)	429 (42.6%)	291 (28.9%)	88 (8.8%)
	0.41	67.1%	62.5%	41.6%	192 (19.1%)	450 (44.7%)	270 (26.8%)	94 (9.4%)
	0.5	56.6%	75.6%	47.9%	162 (16.1%)	544 (54.1%)	176 (17.5%)	124 (12.3%)
	0.53	47.6%	82.2%	51.5%	136 (13.5%)	592 (58.9%)	128 (12.7%)	150 (14.9%)
	0.6	33.2%	90.4%	57.9%	95 (9.4%)	651 (64.7%)	69 (6.9%)	191 (19.0%)
	0.7	12.2%	97.1%	62.5%	35 (3.5%)	699 (69.5%)	21 (2.1%)	251 (24.9%)
MLP	0.3	45.8%	77.5%	44.7%	131 (13.0%)	558 (55.5%)	162 (16.1%)	155 (15.4%)
	0.4	43.4%	80.1%	46.4%	124 (12.3%)	577 (57.4%)	143 (14.2%)	162 (16.1%)
	0.41	44.4%	79.7%	46.5%	127 (12.6%)	574 (57.1%)	146 (14.5%)	159 (15.8%)
	0.5	40.6%	81.1%	46.0%	116 (11.5%)	584 (58.1%)	136 (13.5%)	170 (16.9%)
	0.53	41.3%	81.7%	47.2%	118 (11.7%)	588 (58.5%)	132 (13.1%)	168 (16.7%)
	0.6	40.6%	83.6%	49.6%	116 (11.6%)	602 (59.8%)	118 (11.7%)	170 (16.9%)
	0.7	35.0%	85.4%	48.8%	100 (9.9%)	615 (61.1%)	105 (10.5%)	186 (18.5%)

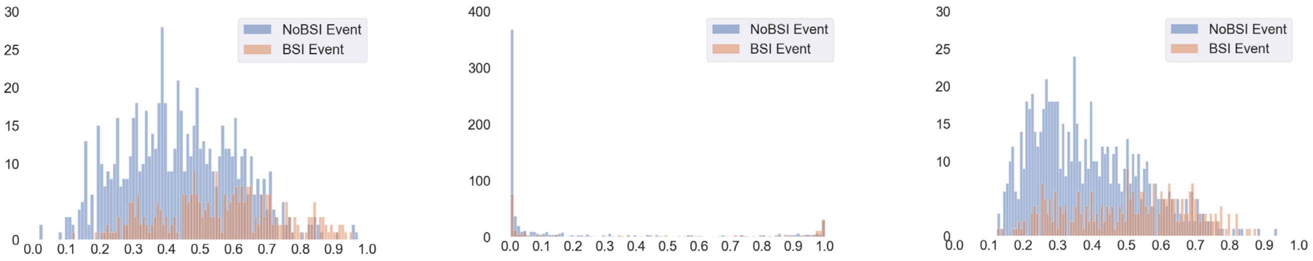

(**a**) (**b**) (**c**)

Figure S1. Model Performance statistics for: (a) logistic regression model, (b) multi-layer perceptron model, (c) support vector machine model.

References

1. Lee, K.H.; Dong, J.J.; Jeong, S.J.; Chae, M.-H.; Lee, B.S.; Kim, H.J.; Ko, S.H.; Song, Y.G. Early Detection of Bacteraemia Using Ten Clinical Variables with an Artificial Neural Network Approach. *J. Clin. Med*. **2019**, *8*, 1592.
2. Roimi, M.; Neuberger, A.; Shrot, A.; Paul, M.; Geffen, Y.; Bar-Lavie, Y. Early diagnosis of bloodstream infections in the intensive care unit using machine-learning algorithms. *Intensive Care Med.* **2020**, *46*, 454–462.
3. Park, H.J.; Jung, D.Y.; Ji, W.; Choi, C.M. Detection of Bacteremia in Surgical In-Patients Using Recurrent Neural Network Based on Time Series Records: Development and Validation Study. *J. Med. Internet Res.* **2020**, *46*, e19512.
4. Mahmoud, E.; Dhoayan, M.; Bosaeed, M.; Johani, S.; Arabi, Y.M. Developing Machine-Learning Prediction Algorithm for Bacteremia in Admitted Patients. *Infect. Drug Resist.* **2021**, *14*, 757–765.
5. Tsai, C.M.; Lin, C.R.; Zhang, H.; Chiu, I.M.; Cheng, C.Y.; Yu, H.R.; Huang, Y.H. Using Machine Learning to Predict Bacteremia in Febrile Children Presented to the Emergency Department. *Diagnostics* **2020**, *10*, 307.
